# Exopolysaccharide Produced by *Lactobacillus paracasei* IJH-SONE68 Prevents and Improves the Picryl Chloride-Induced Contact Dermatitis

**DOI:** 10.3390/molecules24162970

**Published:** 2019-08-16

**Authors:** Masafumi Noda, Nasrin Sultana, Ikue Hayashi, Mitsuhiro Fukamachi, Masanori Sugiyama

**Affiliations:** 1 Department of Probiotic Science for Preventive Medicine, Graduate School of Biomedical and Health Sciences, Hiroshima University, Kasumi 1-2-3, Minami-ku, Hiroshima 734-8551, Japan; 2 Central Research Laboratory, Graduate School of Biomedical and Health Sciences, Hiroshima University, Kasumi 1-2-3, Minami-ku, Hiroshima 734-8551, Japan; 3 Asahi Kohsan Co., Ltd., Shinjuku 1-1-14, Shinjuku-ku, Tokyo 160-0022, Japan

**Keywords:** exopolysaccharide, *Lactobacillus paracasei*, contact dermatitis, lactic acid bacteria

## Abstract

Allergic disease is one of the most important and common health problems worldwide. We have previously demonstrated that a fig leaf-derived lactic acid bacterium *Lactobacillus* (*Lb.*) *paracasei* IJH-SONE68 produces a novel exopolysaccharide (EPS). Furthermore, we have shown that the EPS inhibits the catalytic activity of hyaluronidase (EC 3.2.1.36) promoting inflammatory reactions. To evaluate the anti-allergy and anti-inflammatory effects of the EPS, in the present study, we employed the picryl-chloride-induced delayed-type (type IV) allergy model mice, which is used to evaluate the contact dermatitis. Oral administration of the EPS was observed to reduce the ear swelling in the model mice. We also observed that the overexpression of ear interleukin-4 (T helper (Th) 2 cytokine) mRNA and the increase in serum immunoglobulin E (IgE) are repressed. However, the expression of interferon-γ (Th1 cytokine) was not accelerated in all of the allergen-challenged model mice. The improvement may be responsible for the Th2 downregulation rather than the Th1 upregulation. In addition, the symptom of immediate-type (type I) allergy model mice was improved by oral administration of the IJH-SONE68 cell (data not shown). We can conclude that the IJH-SONE68-derived EPS is useful to improve the type I and IV allergies including atopic dermatitis.

## 1. Introduction

Lactic acid bacteria (LABs) are typical probiotics that are an order of gram-positive and generally non-pathogenic bacteria. Probiotics are defined as living microorganisms that confer health benefits to hosts such as humans [1].

LABs produce a large amount of lactic acid, sometimes together with acetate or ethanol [2]. Some LAB strains, which produce bacteriocin and functional substances, have health benefits for humans [3,4]. Mechnikov of the Pasteur Institute proposed the theory that aging is caused by toxic bacteria in the gut and that lactic acid could prolong life. He presumed that when *Lactobacillus* (*Lb.*) *delbrueckii* subsp. *bulgaricus* (generally called *Lb. bulgaricus*) is orally administrated, the bacterium inhibits the growth of intestinal toxin-producing bacteria [5]. That hypothesis has already been rejected; however, various studies have clarified that many LAB strains do bring potential health benefits [4,6,7,8].

We have isolated many kinds of plant-derived LABs, which will be useful for preventive medicine [9,10,11]. In our library composed of the plant-derived LABs of more than 700 strains, we found that *Lb. paracasei* IJH-SONE68, isolated from a fig leaf, produces neutral and acidic exopolysaccharides (EPSs) [12]. In the previous study, we have found by nuclear magnetic resonance (NMR) analysis that the neutral EPS mainly consists of α-1,6 linked glycan chains made of *N*-acetylglucosamine (GlcNAc), demonstrating that the neutral EPS has a novel structure that has never been reported [12]. On the other hand, the acidic EPS is consisting of primarily mannose. Furthermore, it was demonstrated that the neutral EPSs strongly inhibit the catalytic activity of hyaluronidase (EC 3.2.1.35) [12].

Hyaluronidase catalyzes the hydrolysis of hyaluronic acid as a main constituent of the extracellular matrix. The enzyme promotes inflammation by hydrolyzing hyaluronic acid under inflammatory conditions [13,14,15,16]. The inhibitory effect on hyaluronidase has been observed in some anti-allergic and anti-inflammation agents [17,18]. That is, the substance, which displays the hyaluronidase-inhibitory effect, might be useful to prevent or improve the allergy and inflammation diseases.

Picryl chloride (PiCl), which has been utilized for a long time, is a simple chemical hapten reagent [19] used to prepare the delayed type hypersensitivity model animals [20,21]. Therefore, we used PiCl as the hapten to prepare the hypersensitivity model. In the present study, we use PiCl-induced delayed-type allergy model mice to show that the IJH-SONE68-derived EPS improves and prevents type I and type IV allergic diseases.

## 2. Results

### 2.1. Preventive Effect of IJH-SONE68-Derived EPS Observed on Delayed-Type Allergy Mouse Model

**Figure 1:**
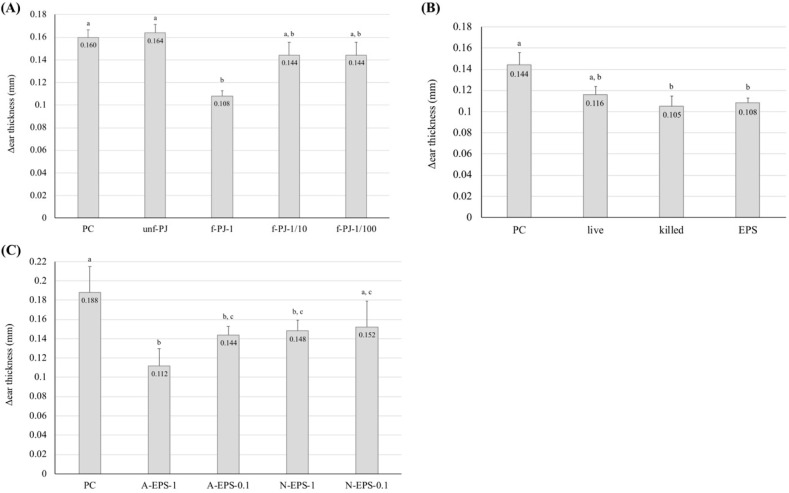
Observed improvement in the PiCl-induced ear inflammation by oral administration of (**A**) IJH-SONE68-fermented pineapple juice, (**B**) the cell suspension or EPS fraction, and (**C**) purified neutral and acidic EPSs. Ear thickness was measured just before and 24 h after the challenge. Abbreviations used in the figure: PC, positive control (without treatment); unf-PJ, unfermented pineapple juice; f-PJ-1, fermented pineapple juice (undiluted); f-PJ-1/10, 10-fold diluted fermented pineapple juice; f-PJ-1/100, 100-fold diluted fermented pineapple juice; live, living cells; killed, heat-killed cells; EPS, extracted EPS fraction containing both neutral and acidic EPSs (1 mg/mL); A-EPS-1, acidic EPS (1 mg/mL); A-EPS-0.1, acidic EPS (0.1 mg/mL); N-EPS-1, neutral EPS (1 mg/mL); N-EPS-0.1, neutral EPS (0.1 mg/mL). Data are indicated by mean ± S.E. of 4–5 mice. Different characters described on the top of the bars indicate statistically significant differences between means of values obtained in different group (the Tukey–Kramer test, *p* < 0.05).

In this study, pineapple juice supplemented with sake lee was used as the culture medium for the IJH-SONE68 strain. Figure 1A shows that oral administration of the LAB-fermented pineapple juice (the undiluted broth) significantly (*p* < 0.01) repressed the thickness of the swollen ear on the PiCl-induced delayed-type allergy model mice, whereas the administration of sterile distilled water did not. Although the observed repression was not significant in the 10- or 100-fold diluted fermented pineapple juice group, the swelling was definitely decreased when compared with the positive control group. Understandably, the pineapple juice without fermentation did not repress the ear swelling.

In a subsequent experiment, we evaluated whether the LAB cells and EPS fraction (the unseparated sample of neutral and acidic EPSs) prevent or repress the PiCl-induced delayed-type allergy (Figure 1B). Under the present experimental conditions, the cell suspension adjusted to 1 × 10^12^ cfu/mL and the EPS solution (1 mg/mL) were employed. Oral administration of the heat-killed cells significantly repressed the inflammatory reaction, whereas that of the living cells did not, suggesting that the IJH-SONE68-derived EPS is the functional molecule with regard to delayed-type allergy. We also examined whether the functional molecule is the neutral or acidic EPS (1.0 or 0.1 mg/mL solution). Figure 1C shows that acidic EPS effectively prevents the inflammatory reaction. The positive effect was significantly reduced by dilution of the EPS fraction.

### 2.2. Deference in the Expression Level of Inflammatory Cytokines

**Figure 2:**
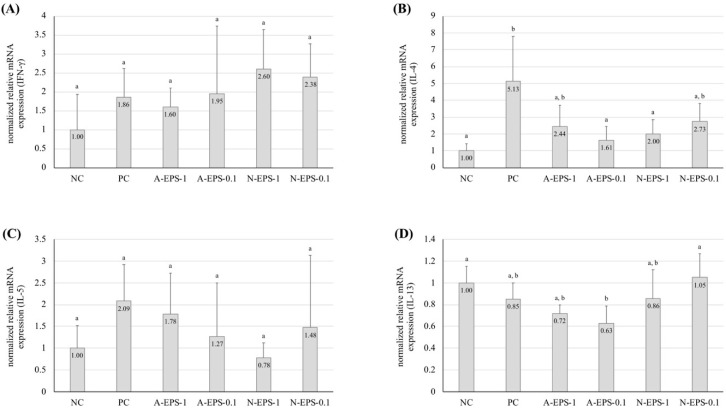
Effects of oral administration of IJH-SONE68-derived neutral and acidic EPSs on the mRNA level of IFN-γ (**A**), IL-4 (**B**), IL-5 (**C**), and IL-13 (**D**) expressed in the ear homogenates at 24 h after the challenge. Each expression level was normalized to that of the β-actin gene as a reference. Abbreviations used in the figure: NC, negative control (without inflammation); PC, positive control (without treatment); A-EPS-1, acidic EPS (1 mg/mL); A-EPS-0.1, acidic EPS (0.1 mg/mL); N-EPS-1, neutral EPS (1 mg/mL); N-EPS-0.1, neutral EPS (0.1 mg/mL). Data are indicated by mean ± S.D. Different characters described on the top of the bars indicate statistically significant differences between means of values obtained in different group (the Tukey–Kramer test, *p* < 0.05).

To understand the preventive mechanism against the contact dermatitis model observed with administration of the IJH-SONE68-derived EPSs, the mRNA expression levels of T helper (Th) 1-type (interferon (IFN)-γ) and Th2-type (interleukin (IL)-4, 5, and 13) inflammatory cytokines in ear tissue were measured using quantitative reverse transcriptional (qRT)-PCR analysis. Figure 2 shows that the IFN-γ expression in all of the allergen-challenged group was approximately 2-fold higher than that in the unchallenged group, but the differences were not significant (Figure 2A). In contrast, the relative expression level of IL-4 was significantly increased in the positive control group, and the IL-4 increase was repressed by the administration of EPS, except for the 10-fold diluted neutral EPS (Figure 2B). The expression levels of IL-5 and IL-13 were not distinguishable between the positive control and the EPS-administration groups (Figure 2C,D).

### 2.3. Relation Between the Serum Immunoglobulin E (IgE) Level and Ear Inflammation

**Figure 3:**
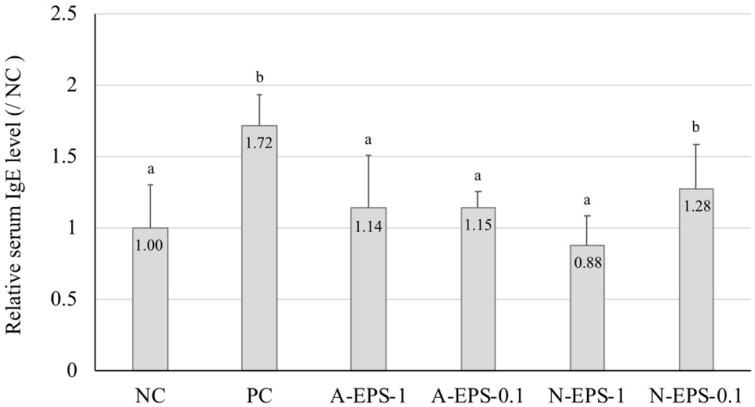
Effect of oral administration of IJH-SONE68-derived neutral and acidic EPSs on the serum IgE concentration at 24 h after the challenge. Abbreviations used in the figure: NC, negative control (without inflammation); PC, positive control (without treatment); A-EPS-1, acidic EPS (1 mg/mL); A-EPS-0.1, acidic EPS (0.1 mg/mL); N-EPS-1, neutral EPS (1 mg/mL); N-EPS-0.1, neutral EPS (0.1 mg/mL). Different characters described on the top of the bars indicate statistically significant differences between means of values obtained in different group (the Tukey–Kramer test, *p* < 0.05).

The IgE level in the serum of type I allergic patients, which are known as pollinosis and allergic rhinitis, is significantly higher than in that of healthy persons [22]. In the present study, we measured the IgE level linked to an inflammatory reaction of the ear (Figure 3). The serum IgE level in the positive control group was higher than that in the negative control group. However, the IgE level increased by contact dermatitis was repressed in the EPS-administration group, except for the 10-fold diluted neutral EPS group. This result corresponds with that obtained for the IL-4 expression level, showing that the ear inflammation correlates strongly with the IgE level.

### 2.4. Histological Changes in Auricula

**Figure 4:**
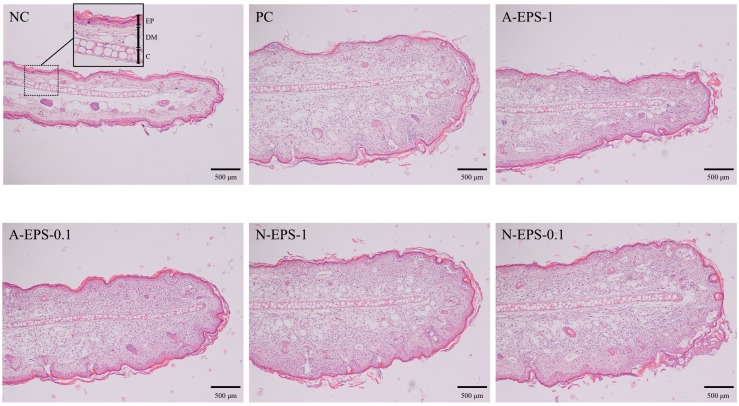
Effect of oral administration of IJH-SONE68-derived neutral and acidic EPSs to the ear lobe of PiCl-induced contact dermatitis mice. A representative tissue section of the ear lobe was stained with hematoxylin and eosin. Abbreviations used in the figure: NC, negative control (without inflammation); PC, positive control (without treatment); A-EPS-1, acidic EPS (1 mg/mL); A-EPS-0.1, acidic EPS (0.1 mg/mL); N-EPS-1, neutral EPS (1 mg/mL); N-EPS-0.1, neutral EPS (0.1 mg/mL); EP, epidermis layer; DM, dermis layer; C, cartilage. Scale bar = 500 μm.

The paraffin-embedded ear tissue sections from each mouse were subjected to histological comparison (Figure 4). As a result of the allergic reaction induced by PiCl, the dermis layer in the positive control group was thicker than that in the negative control group. In the group with EPS administration, the thickness of the dermis layer was gradually restored in proportion to the inflammation recovery. The partial damage of the epidermis layer, which is stained a deep red color in the external layer, was observed, but not in the negative control. That is, the damage was recovered by EPS treatment.

### 2.5. Recovery from the Delayed-Type Allergy by Direct Application of the IJH-SONE68-Derived EPS to the Mouse Ear

**Figure 5:**
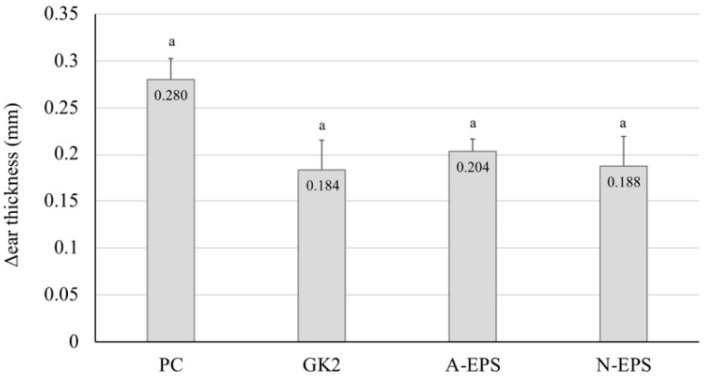
Improvement in PiCl-induced contact dermatitis by application of IJH-SONE68-derived neutral and acidic EPSs to the mouse ear. Ear thickness was measured at 0 h and 24 h after the allergen challenge. A 20-μL aliquot of each sample (1 mg/mL) was applied to the ear lobe 2 h after the challenge. Data are indicated by mean ± S.D. of 5 mice. Abbreviations used in the figure: PC, positive control (without treatment); GK2, dipotassium glycyrrhizinate (1 mg/mL); A-EPS, acidic EPS (1 mg/mL); N-EPS-1, neutral EPS (1 mg/mL). Different characters described on the top of the bars indicate statistically significant differences between means of values obtained in different group (the Tukey–Kramer test, *p* < 0.05).

To confirm whether the EPS has a therapeutic effect on the delayed-type allergy model mice, we tested direct application of the IJH-SONE68-derived EPS to the mouse earlobe (Figure 5). The result demonstrates that the ear thickness of the tested groups became slightly thinner than that of the untreated group, but a significant difference was not observed. However, the application of dipotassium glycyrrhizinate or the neutral EPS to the earlobe tended to decrease (*p* = 0.077 and 0.094, respectively) the ear thickness.

## 3. Discussion

**Figure 6:**
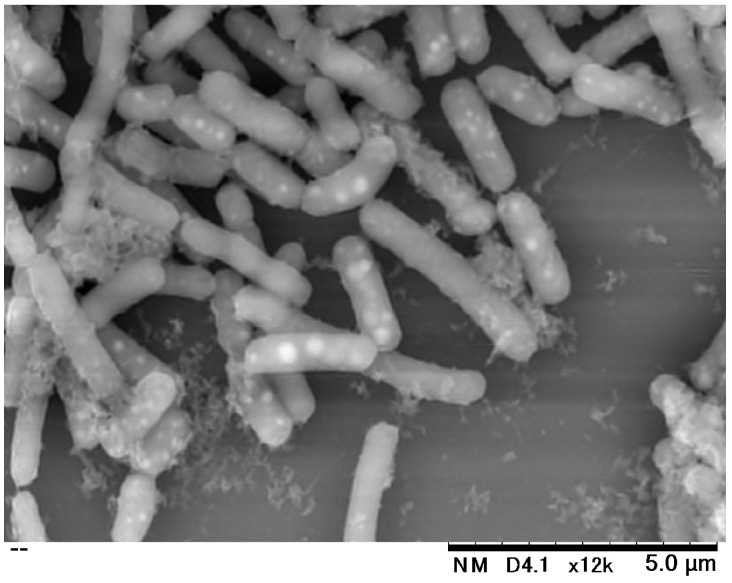
Scanning electron microscopic image of the *Lb. paracasei* IJH-SONE68 cells.

Several strains of *Lb. paracasei*, which has been used to manufacture dairy products, have been reported to be effective for the immune-modulation and the reduction of allergic symptoms. With respect to the health benefits, same species have been reported as follows: *Lb. paracasei* MCC1849 enhances the expression of antigen-specific IgA [23], *Lb. paracasei* LP33 is effective against allergic rhinitis [24], and *Lb. paracasei* KW3110 reduces allergic inflammation in patients with cedar pollinosis [25]. We demonstrated through the present study that the anti-allergic effect of the IJH-SONE68 strain is brought by the EPSs rather than by cellular components. The effectiveness of the IJH-SONE68 strain against delayed-type allergy was also observed in both living and heat-killed cells. That is, the EPSs might not be completely liberated from the bacterial cell wall even in heat-killed cells. The SEM image shows that the EPS adheres to the cell surface of the IJH-SONE68 strain (Figure 6).

The type I allergy, which is an immediate allergy, includes pollinosis and allergic rhinitis as typical disorders [22]. In this allergy type, the specific invading antigens (allergens) promote proliferation of the Th2 cell, which releases IL-4, resulting in antigen-specific IgE production. When the antigens are recognized by the IgE molecule bound to the mast cell through the specific receptor, the mast cell releases chemical mediators, like histamine, that induce immediate allergic reactions.

The type IV allergy, which is a delayed allergy, is thought to be mainly associated with activation of the Th1 cell. If the antigen is present, the proliferated Th1 cell releases cytokines to induce granulocyte infiltration and the accumulation of macrophages. PiCl is an agent that confers the picryl group to skin proteins [26]. The PiCl–protein conjugate acts as a hapten and induces the allergic delayed-type allergy, which is classified as a type IV allergy and is historically mediated by specific T lymphocytes.

In the IFN-γ or IFN-γ receptor–deficient mice, the contact dermatitis allergic reactions were also observed as in the wild-type mice [27,28,29,30]. Furthermore, the contact allergy was suppressed in the knockout mice with a signal transducer and activator of the transcription 6 (Stat6) gene, which participates in the signal transduction of IL-4 and IL-13 [31]. Especially in the atopic dermatitis, it has been shown that IL-4 production by Th2 cells is predominant at the initiation phase, but IFN-γ production by Th1 cells is predominant at the late and chronic phases [32]. Judging from these reports and the results of our present study, the Th2 cytokines may function on the delayed-type allergy. It has been also observed that the serum IgE and tissue IL-4 are increased in the contact dermatitis model mice [33]. In the present study, we demonstrated that the expression of IL-4 and serum IgE were accelerated in the PiCl-induced delayed-type allergy, and the allergic inflammation was significantly repressed by EPS administration.

In general, the observed improvement of allergic symptoms upon oral administration of the bacterial cells is due to the inhibition of Th2 cell proliferation via Th1 cell induction by antigen-presenting cells that recognize the bacterial cell as an antigen [6]. By this mechanism, Th1 acceleration causes the repression of Th2 induction. As a result, the progression of an allergic reaction may be controlled. However, our result shows that IJH-SONE68-derived EPS administration does not accelerate the expression of IFN-γ but represses that of IL-4. This result indicates that the improvement observed with IJH-SONE68-derived EPS in the delayed-type allergy model mice is due to Th2 downregulation rather than Th1 upregulation. In addition, oral administration of the IJH-SONE68 cell has been confirmed to improve the allergic reactions observed in the immediate-type allergy model mice (data not shown). IL-4 is essential cytokine to induce the production of IgE that potentially worsens the symptoms of atopic dermatitis, which is known as a mixed model of the type I and IV allergies [34]. Therefore, the IJH-SONE68-derived EPS may be useful to improve the type I and IV allergic reactions including atopic dermatitis.

Since EPS is composed of a long chain of monosaccharide units, the macromolecule does not seem to be absorbed into the blood through the intestinal cell wall without digestion by intestinal microorganisms and enzymes. The EPS produced by *Lb. delbrueckii* subsp. *bulgaricus* OLL1073R-1 has been reported to enhance the natural killer (NK) cell activity in mice [35]. The stimulating effect did not appear in the mice lacking myeloid differentiation factor 88 (MyD88), which is an adaptor molecule for the inflammatory signaling pathways of toll-like receptors (TLRs) [36]. In the MyD88-knockout mouse, it has been reported that the reactivity of TLR-2, TLR-4, TLR-5, TLR-7, and TLR-9 against each corresponding ligand is lost [37]. Tohno et al. have reported that a TLR-4 homologue, RP105, recognizes not only lipopolysaccharide (LPS) known as ligand of TLR-4, but also the phosphorylated EPS from *Lactococcus* (*Lc.*) *lactis* subsp. *cremoris* GCL1176 [38]. Judging from the study, TLR-4 is likely to a receptor for the IJH-SONE68-derived EPSs and that LPS and EPS can induce the production of some cytokines via same pathway. In addition, although IL-4 produced by Th2 cells induces the IgE production from B cell, one of the LPS derivatives, monophosphoryl lipid A has been found to inhibit the production of antigen-specific IgE [39,40]. On the other hand, Gotoh et al. have reported that the anti-inflammatory effect of *Lc. lactis* subsp. *cremoris* FC-derived EPS observed on the dermatitis model mice seems to be due to the modulation of the systemic immune system through a Peyer’s patch-mediated mechanism [41]. However, the inflammation of the mice model using 2,4,6-trinitro-1-chlorobenzene (TNCB) was improved by the oral administration of the EPS without IgE decrease. Some LAB strains improve the murine dermatitis model in a manner independent of the IgE level [42,43]. Therefore, the hypersensitivity-improving effect of EPS might be different with and without IgE changes, suggesting that each receptor may recognize separately the individual tertiary structure of EPS.

In the present study, the IJH-SONE68-derived EPSs are found to exhibit anti-inflammatory activity by direct application to the mouse ear. The predicted mechanism may be distinguished from the improvement observed with oral administration. The EPS applied to the skin is predicted to permeate the skin, and the macromolecule may inhibit the enzymatic activity of hyaluronidase, which stimulates the inflammatory reaction. In the previous study, we evaluated the hyaluronidase-inhibitory activity of several LAB-derived EPSs, sodium cromoglicate, dipotassium glycyrrhizinate, and ketotifen fumarate, which are known as substances repressing the type I allergy [12,44,45]. Among those substances, ketotifen fumarate was found not to have detectable anti-hyaluronidase activity [44]. This result is presumed to be due to the different pharmacological mechanisms: sodium cromoglicate and dipotassium glycyrrhizinate inhibit the release and production of a chemical mediator, respectively [46,47], but the mechanism of ketotifen fumarate is inhibition of the histamine receptor [48,49]. Since the hyaluronidase inhibitory activity correlates with histamine-release inhibition [17,18], the therapeutic effect of the IJH-SONE68-derived EPSs may be involved in the inhibition of a chemical mediator release rather than act as histamine antagonist, if the EPS was digested from macromolecule to small molecules.

In the previous study [12], the main structure of the IJH-SONE68-derived neutral EPS has been determined to be an α-1,6-linked GlcNAc polymer. In addition, the acidic EPS consists of glucose, mannose, galactose, and rhamnose at a ratio of 10:170:2:1. We have also determined the predicted EPS-biosynthesizing gene clusters located on the IJH-SONE68 chromosomal DNA [12]. But, at this moment, we do not elucidate a relationship between the chemical structure of EPS and the anti-inflammatory activity. To know it, the chemical structures of other lactic acid bacterial EPS are necessary to be provided.

LABs and a genus *Bifidobacterium* are known to confer health benefits to humans. In addition to the bacterial cells, LAB-derived EPS provides some health benefits [12,44,45,50]. The immunomodulatory effect on the J774A.1 macrophage, anti-microbial activities [51], and enhancement of NK cell activity [35] of EPSs have also been reported to confer the health benefits to humans. Through animal experiments, the EPS has been shown to be effective for immune modulation [51], to exhibit anti-tumor activity [52], and to decrease blood cholesterol [53]. Recently, oral administration of the EPS-producing LAB has been expected to be used in inflammatory bowel disease therapy [54].

## 4. Materials and Methods

### 4.1. Media and Growth Conditions

A de Man, Rogosa, and Sharpe (MRS) medium (Merck KGaA, Darmstadt, Germany) was used as a seed culture medium for *Lb. paracasei* IJH-SONE68. A modified semi-defined medium (SDM) supplemented with a vitamin mixture (0.2 *v*/*v* %) and trace element solution (0.1 *v*/*v* %) instead of yeast nitrogen bases was used as a main culture medium [55,56,57].

The IJH-SONE68 cell mass used for oral administration was prepared by the following method: the LAB strain was grown in the modified SDM at 28 °C for 48 h. The cell mass, which was obtained by centrifugation of the cultured broth, was washed and diluted with sterile distilled water to 1 × 10^12^ cfu/mL. If necessary, the washed cell mass was heat-treated in an autoclave (at 121 °C for 20 min). Prior to cultivation of the LAB strain using pineapple juice supplemented with 1 (*w*/*v*) % spray-dried sake lees, the culture medium was adjusted to pH 6.0 with NaOH and sterilized at 100 °C for 10 min. If necessary, the pineapple juice fermented by the IJH-SONE68 strain was 10- or 100-times diluted by sterile distilled water.

### 4.2. Purification of EPSs

The stock culture of the IJH-SONE68 strain stored at −80 °C was inoculated into a fresh MRS medium. After the two-day standing cultivation at 28 °C, the cells collected by centrifugation were washed with a sterilized phosphate-buffered saline (PBS: pH 7.4) and resuspended into the same volume of PBS. The resulting cell suspension was inoculated into a modified SDM with a 0.2% volume and grown at 28 °C for 2 days. After the cultivation, at a final concentration, a 4% (*v*/*v*) volume of trichloroacetic acid (TCA) solution was added to the culture broth. The culture supernatant obtained by centrifugation was added with the same volume of acetone. The resulting precipitate containing the EPS was dissolved in a 50 mM Tris–HCl buffer (pH 8.0). After treatment with nuclease and protease, the neutral and acidic EPSs were separated by column chromatography using the anion exchange resin (TOYOPEARL DEAE-650M, Tosoh Bioscience, Tokyo, Japan) as described previously [44]. Each of the purified EPSs was stored at −20 °C until use. Prior to the oral administration, the EPS samples were dialyzed against sterile distilled water by using Amicon Ultra (MWCO = 10 kDa, Merck Millipore Ltd., Carrigtwohill, Co., Cork, Ireland), and the concentrations (1.0 and 0.1 mg/mL) were adjusted with the sterile distilled water.

### 4.3. Experimental Animals

Male specific pathogen free (SPF) BALB/cAJcl seven-week-old mice were purchased from CLEA Japan, Inc. (Tokyo, Japan), and divided into 4–6 experimental groups of 5 mice each after a 1-week acclimation period. The mice were housed in plastic cages under 20–26 °C, 40–60% humidity, and 12-h light/12-h dark conditions. The mice were freely fed with an MF diet (Oriental Yeast Co., Ltd., Tokyo, Japan) and could freely access water. The animal experiment was conducted in accordance with the Guidelines for the Care and Use of Laboratory Animals of Hiroshima University. The mice were distinguished by marking their tails with different colors using felt markers (Animal Marker, Muromachi Kikai Co., Ltd., Tokyo, Japan). After the experimental period, the mice were euthanized via inhalation of anesthesia with isoflurane. The experimental protocol for the animal study was approved by the committee of the Research Facilities for Laboratory Animal Science of Hiroshima University (approval number A17-122).

### 4.4. Evaluation of Anti-Allergic Inflammatory Activity on a PiCl-Induced Delayed-Type Allergy Mouse Model

The after a one-week acclimation period, we began orally administering a 200-μL aliquot of each sample to mice every day during the experimental period using oral feeding needle. After 6 days, the mice were immunized with 7% (*w*/*v*) PiCl dissolved into an acetone: sunflower oil mixture (4:1). In this experiment, a 20-μL portion of the 7% (*w*/*v*) PiCl solution was applied to each footpad of the mice. One hundred microliters of the PiCl solution was also applied to the breasts and backs of the mice. One week later, 20-μL of 1% (*w*/*v*) PiCl solution was applied to both the front and back of the ear. The thickness of ear was measured just before and 24 h after the allergen challenge using a vernier scale (resolution 0.02 mm), and the averaged increment of ear thickness (Δ ear thickness) in the negative control group was only 0.0056 mm, showing that the value is much smaller than the measurement limit (0.02 mm). Therefore, we conclude that the employment of the PiCl-induced mice model is suitable to evaluate the anti-hypersensitive effects of samples. The Δ ear thickness was assessed using the Tukey–Kramer test [58]. The statistical analyses were done using the SPSS 17.0 software (IBM Corporation, New York, NY, USA).

**Figure 7:**
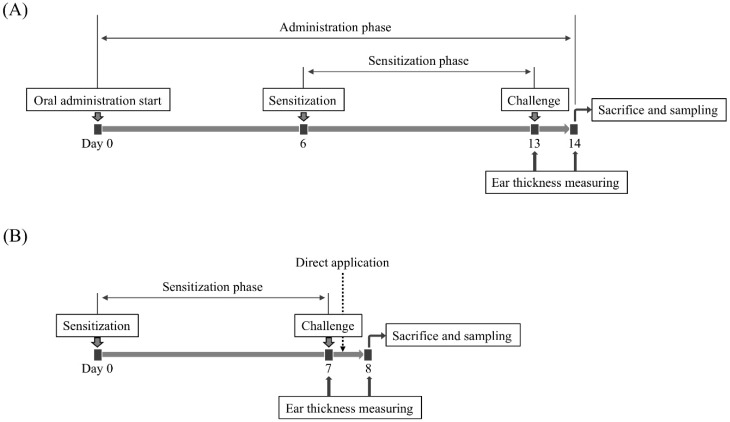
Outline of the animal experiment. Mice were sensitized with PiCl 7 days before the allergen challenge. (**A**) After a one-week acclimation period, 200 μL of each sample was orally administered once a day during the experimental period until sacrifice. At day 13, the mice were challenged via the ear with PiCl. After 24 h, the mice were sacrificed. (**B**) Instead of oral administration, each sample was directly applied to the ear lobe after 2 h of the allergen challenge.

To evaluate the direct effect of EPSs on the skin, dipotassium glycyrrhizinate, which is an anti-inflammatory compound, was used as a positive control. In the procedure, 2 h after the challenging, 20 μL of EPS solution or dipotassium glycyrrhizinate (1 mg/mL each) was applied to the back and front surfaces of the ears of mice without an oral administration phase. The ear thickness was measured after an additional 20 h of breeding. The animal experiment is summarized in Figure 7.

The EPS samples were dissolved into sterile distilled water. If necessary, the EPS samples and the fermented pineapple juice were diluted with sterile distilled water.

### 4.5. RNA Extraction and qRT-PCR Analysis

Total RNA was extracted from each mouse ear using NucleoSpin RNA II (Macherey-Nagel GmbH & Co. KG, Düren, Germany) according to the manufacturer’s instruction manual. The RNA sample was reverse transcribed using the ReverTra Ace qPCR RT master mix with gDNA remover (Toyobo, Osaka, Japan) according to the manufacturer’s instruction manual. The qRT-PCR was conducted on the PikoReal real-time PCR system (Thermo Fisher Scientific, Waltham, MA, USA) using the KAPA SYBR Fast qPCR Kit (Kapa Biosystems, Wilmington, MA, USA). The qPCR was conducted under the following conditions: an initial 30 s at 95 °C, followed by 40 cycles of 5 s at 95 °C and 30 s at 60 °C. The relative transcriptional levels, which were normalized to a reference gene (β-actin), of the IL-4, 5, 13, and IFN-γ were analyzed using the ΔΔCT method. The primers used in this experiment are as follows: 5′-GGGACGACATGGAGAAGA-3′ and 5′-CATACAGGGACAGCACAG-3′ for β-actin; 5′-TCTCGAATGTACCAGGAGC CATATC-3′ and 5′-AGCACCTTGGAAGCCCTACAGA-3′ for IL-4; 5′-GAGAAGTG TGGCGAGGAG-3′ and 5′-GGGGTTTTTGCATCTGT-3′ for IL-5; 5′-GGAGAGGCA GGGAGGAGG-3′ and 5′-GTGGGGTGGGGGTGTTG-3′ for IL-13; and 5′-GAGGAA CTGGCAAAAGGATG-3′ and 5′-GCTGATGGCCTGATTGTCTT-3′ for INF-γ.

### 4.6. Measurement of Serum IgE Level

Serum was extracted from blood samples collected from the mice of each group using a Separapid microtube S (Fuchigami Kikai, Kyoto, Japan). The IgE concentration in each serum was measured using the Mouse IgE ELISA development kit (HRP) (Mabtech, Nacka Strand, Sweden), according to the manufacture’s instruction manual.

### 4.7. Histological Analysis

Mouse ears were obtained 24 h after the allergen challenge. The ears were fixed with 10% (*v*/*v*) formalin and embedded in a paraffin block. The histopathological tissue section was stained with hematoxylin-eosin (HE).

### 4.8. Scanning Electron Microscopy (SEM) Analysis

The IJH-SONE68 cells were fixed with 2% (*w*/*v*) glutaraldehyde for 2 h at room temperature. The PBS-washed cells were used for SEM analysis. The SEM image was observed and captured at a magnification of 12,000×, with an acceleration voltage of 15 kV, using a Hitachi Tabletop Microscope TM3030Plus (Hitachi High-Technologies Corp., Tokyo, Japan).

## 5. Conclusions

It has been known that IgE worsens the symptoms of atopic dermatitis as a mixed model of the type I and IV allergies. In the present study, we observed that the oral administration of the IJH-SONE68-derived EPS represses the expression of IL-4 and serum IgE accelerated in the PiCl-induced delayed-type allergy model mice, suggesting that the IJH-SONE68-derived EPS is effective to improve both types of allergic reactions. Significantly, the acidic EPS is more effective than the neutral one. The improvement may be responsible for the Th2 downregulation rather than Th1 upregulation. It is interesting and significant to understand the relationship between the chemical structure and physiological functions of the EPS.

## Abbreviations

EPS	exopolysaccharide
GlcNAc	*N*-acetylglucosamine
HE	hematoxylin-eosin
IFN	interferon
Ig	immunoglobulin
IL	interleukin
LAB	lactic acid bacterium
*Lb.*	*Lactobacillus*
MRS	de Man, Rogosa, and Sharpe
NMR	nuclear magnetic resonance
PBS	phosphate-buffered saline
PiCl	picryl chloride
qRT-PCR	quantitative reverse transcription PCR
SDM	semi-defined medium
SEM	scanning electron microscopy
SPF	specific pathogen free
TCA	trichloroacetic acid
Th	T helper
TLR	toll-like receptor
